# A scoping review of studies using observational data to optimise dynamic treatment regimens

**DOI:** 10.1186/s12874-021-01211-2

**Published:** 2021-02-22

**Authors:** Robert K. Mahar, Myra B. McGuinness, Bibhas Chakraborty, John B. Carlin, Maarten J. IJzerman, Julie A. Simpson

**Affiliations:** 1 Biostatistics Unit, Centre for Epidemiology and Biostatistics, Melbourne School of Population and Global Health, University of Melbourne, Parkville, Victoria Australia; 2 Cancer Health Services Research Unit, University of Melbourne Centre for Cancer Research and Centre for Health Policy, Melbourne School of Population and Global Health, University of Melbourne, Parkville, Victoria Australia; 3 Victorian Comprehensive Cancer Centre, Parkville, Victoria Australia; 4 Centre for Eye Research Australia, Royal Victorian Eye and Ear Hospital, Melbourne, Victoria Australia; 5 Centre for Quantitative Medicine, Duke-NUS Medical School, Singapore, Singapore; 6 Department of Statistics and Applied Probability, Faculty of Science, National University of Singapore, Singapore, Singapore; 7 Department of Biostatistics and Bioinformatics, Duke University, Durham, North Carolina USA; 8 Clinical Epidemiology and Biostatistics Unit, Murdoch Children’s Research Institute, Parkville, Victoria Australia; 9 Peter MacCallum Cancer Centre, Parkville, Victoria Australia

**Keywords:** Dynamic treatment regimens, Adaptive treatment policies, Sequential multiple assignment randomised trials, Observational data, Causal inference, Directed acyclic graphs

## Abstract

**Background:**

Dynamic treatment regimens (DTRs) formalise the multi-stage and dynamic decision problems that clinicians often face when treating chronic or progressive medical conditions. Compared to randomised controlled trials, using observational data to optimise DTRs may allow a wider range of treatments to be evaluated at a lower cost. This review aimed to provide an overview of how DTRs are optimised with observational data in practice.

**Methods:**

Using the PubMed database, a scoping review of studies in which DTRs were optimised using observational data was performed in October 2020. Data extracted from eligible articles included target medical condition, source and type of data, statistical methods, and translational relevance of the included studies.

**Results:**

From 209 PubMed abstracts, 37 full-text articles were identified, and a further 26 were screened from the reference lists, totalling 63 articles for inclusion in a narrative data synthesis. Observational DTR models are a recent development and their application has been concentrated in a few medical areas, primarily HIV/AIDS (27, 43%), followed by cancer (8, 13%), and diabetes (6, 10%). There was substantial variation in the scope, intent, complexity, and quality between the included studies. Statistical methods that were used included inverse-probability weighting (26, 41%), the parametric G-formula (16, 25%), Q-learning (10, 16%), G-estimation (4, 6%), targeted maximum likelihood/minimum loss-based estimation (4, 6%), regret regression (3, 5%), and other less common approaches (10, 16%). Notably, studies that were primarily intended to address real-world clinical questions (18, 29%) tended to use inverse-probability weighting and the parametric G-formula, relatively well-established methods, along with a large amount of data. Studies focused on methodological developments (45, 71%) tended to be more complicated and included a demonstrative real-world application only.

**Conclusions:**

As chronic and progressive conditions become more common, the need will grow for personalised treatments and methods to estimate the effects of DTRs. Observational DTR studies will be necessary, but so far their use to inform clinical practice has been limited. Focusing on simple DTRs, collecting large and rich clinical datasets, and fostering tight partnerships between content experts and data analysts may result in more clinically relevant observational DTR studies.

**Supplementary Information:**

The online version contains supplementary material available at 10.1186/s12874-021-01211-2.

## Background

The medical needs of patients with chronic or progressive conditions often evolve over time and the treatments administered to these patients need to be regularly reviewed. Treatment decisions may depend on the dynamics of a number of factors or require continual switching between different treatments. Therefore, making optimal treatment decisions requires information across many time intervals. *Dynamic treatment regimens* (or *regimes*) (DTRs) formalise the multi-stage and dynamic decision problems clinicians often face when treating chronic or progressive conditions [1–5]. A DTR can be thought of as a set of rules describing how treatment could be assigned in response to some dynamically changing factor, for example, treatment response.

A DTR can be defined using *decision rules*, functions that map each patient’s accumulated clinical and treatment history to the subsequent treatment at each treatment decision point. These rules are typically derived from parametric models. An *optimal* decision rule is one that optimises the long-term *value* of the decision, for example, expected overall survival. The values of the decision rules are estimated using statistical methods that can account for time-varying treatment effect mediation and confounding. In order for the estimated treatment effects that inform the decision rules to have a causal interpretation, a number of conditions must be met, which are summarised in the next section.

One real-world example of a decision problem that has been framed and optimised as a DTR is ‘when to begin’ antiretroviral treatment in patients with human immune-deficiency virus (HIV), which is often based on their CD4 count history [6, 7]. The decision to start a patient’s treatment may not be appropriate if it is based only on their most recent clinical history, ignoring whether their CD4 count has been stable or not. Another real-world example of a DTR is ‘how to modify’ prophylaxis for graft-versus-host disease following stem-cell transplantation for blood cancer, when a patient may receive either the standard or an experimental prophylaxis [8, 9]. If the patient subsequently develops acute graft-versus-host disease (i.e., the allocated prophylactic treatment has not been effective) they may then receive either a standard or an experimental salvage treatment. The selection of treatment at each stage is based on a suite of time-varying disease characteristics.

Optimising DTRs relies on estimating the value of the decision rules using data from either *sequential multiple assignment randomised trials* (SMARTs) [1, 10–12], which are designed to randomise and re-randomise participants to different treatments over time conditional on their observed outcomes, or from observational sources such as cohort studies, electronic health records (EHRs), and clinical registries. Estimating optimal DTRs using SMART data provides the highest-quality evidence of regimen efficacy by reducing confounding bias through randomisation. However, SMARTs are more complex to design and implement than standard trial designs and therefore are resource intensive.

A potentially less costly and more operationally feasible alternative is to emulate a ‘target trial’ using existing observational data [6, 13, 14]. However, without treatment randomisation, the causal relationships between the covariates, treatments, outcomes must be carefully considered, and in particular, it is necessary that all relevant confounders are measured to obtain unbiased estimates of the causal effects of interest [1, 14]. Nevertheless, observational data has several potential advantages over trial data. For example, without rigid inclusion criteria and control protocols, observational data may better reflect the heterogeneity of both patient populations and treatment implementation, which may allow a broader range of treatment regimens to be evaluated and therefore represent actual treatment practice better than trial data. Some authors suggest that optimal DTR-based treatment decisions should be estimated using observational data, where possible, before proceeding to the relevant SMART design stage [1, 15]. Indeed for some dynamic treatment regimens, particularly for ‘when to treat’ regimens that involve delayed treatment, it may be neither feasible nor ethical to conduct a randomised trial.

The effective use of observational data to evaluate dynamic treatment decisions has the potential to provide insight into the management of chronic or progressive conditions, yet it is unclear to what extent it is done in practice. This study provides a scoping review [16] to systematically map how observational data have been used to estimate the value of DTRs in practice with the following specific aims:

- ▪ To summarise what medical areas, participant numbers, types of outcomes, and statistical methods have been used in real-world practice.
- ▪ To describe whether key methodological aspects of the real-world applications were considered.
- ▪ To ascertain whether the real-world application was designed more to inform statistical or clinical practice.

The overarching aim was to identify whether any particular domains dominate the literature and why this may be so, in order to understand the potential for evidence regarding DTRs to be developed using observational data, and to identify existing gaps in the methodological quality of published studies.

The remainder of this article proceeds as follows. We first provide terminology and describe a DTR using a simple two-stage example, selected modelling and estimation approaches for DTR-based decision rules, and the necessary conditions for causal inference. We follow by describing the methods and results of the scoping review to explore the context, methods, and reporting of studies which have modelled DTRs using observational data. We follow with a summary of the results, and general discussion and concluding summary of the key concepts.

### Dynamic treatment regimens

#### Concept and notation

**Fig. 1:**
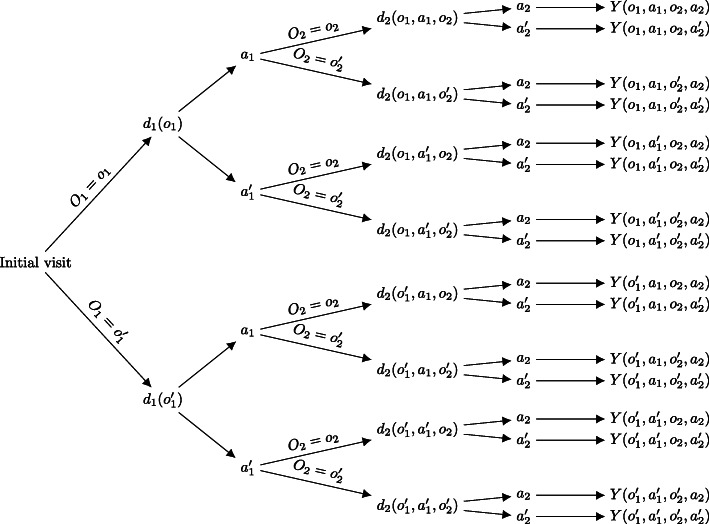
A decision tree containing several possible dynamic treatment regimens (DTRs). Shown are binary covariates (*O*_1_, *O*_2_), binary treatments (*A*_1_, *A*_2_), and a terminal outcome (*Y*) that is a function of patient history. The decisions that map the accumulated patient history to a treatment are represented as the functions *d*_1_(.) and *d*_2_(.)

A simple two-stage, two-treatment scenario that can be formalised using DTRs can be described by the following notation:

$$ {O}_1\to {A}_1\to {O}_2\to {A}_2\to Y $$

where *O*_*k*_ describes the set of prognostic factors available for treatment decision, *A*_*k*_, and the terminal outcome, *Y*, and *k* ∈ *K* = {1, 2} indexes the first and second treatment stages. The accumulated history, *H*_*k*_, includes all covariates and treatments preceding *A*_*k*_. Therefore, in our simple example, *H*_1_ = *O*_1_ and *H*_2_ = {*O*_1_, *A*_1_, *O*_2_}. We follow standard convention and denote random variables and their observed values using upper- and lower-case letters, respectively. DTR models define decision rules *d*_*k*_ as functions that map a patient’s history (*H*_*k*_) to a certain course of action (*A*_*k*_): *d*_*k*_(*H*_*k*_) → *A*_*k*_. Note that a DTR can be generalised to more than two stages and treatments, multiple covariates with different data types, and different outcome types [1]. In Fig. 1, we present a decision tree depicting many possible realised DTRs, where each *O*_*k*_ and *A*_*k*_ are binary variables.

**Fig. 2:**
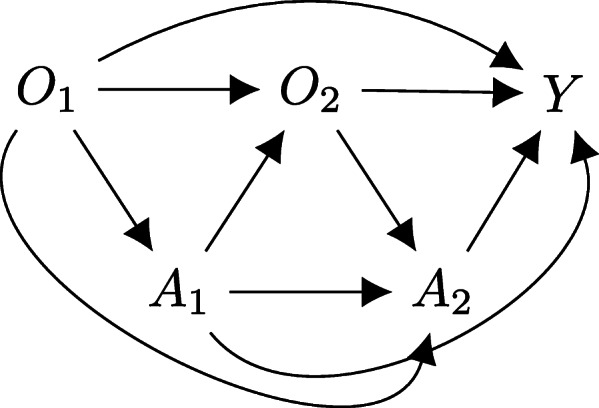
A dynamic treatment regimen (DTR) causal diagram. Covariates (*O*_1_, *O*_2_), treatments (*A*_1_, *A*_2_), and the outcome (*Y*) are each represented by a node with causal relationships shown as directed edges (arrows). Note that the edges are directional and it not possible for a node to cycle back to itself along the graph’s edges, hence it is a directed acyclic graph

The same two-stage scenario presented in Fig. 1 may also be described using a causal diagram or *directed acyclic graph* (DAG) (see Fig. 2). Causal diagrams are a graphical and intuitive way of encoding the causal assumptions that are made when considering how to analyse a problem [14, 17, 18].

#### Modelling dynamic treatment regimens

**Fig. 3:**
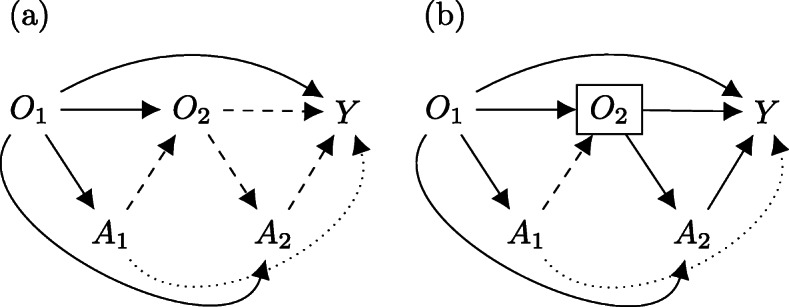
Causal diagram demonstrating effect mediation. Note: **a** Indirect effects of *A*_1_ → *Y* (dashed) mediated by conditioning on *O*_2_ (boxed). **b** Direct effect of *A*_1_ → *Y* (dotted) is not affected

**Fig. 4:**
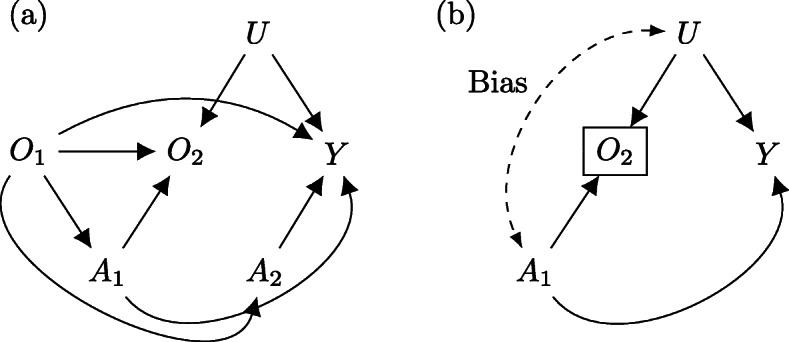
Causal diagram demonstrating collider stratification bias. Note: **a** *O*_2_ does not mediate *O*_1_, and unmeasured confounders (*U*) and *O*_1_ are unrelated. **b** conditioning on *O*_2_ (boxed) may induce collider stratification bias (dashed) between *A*_1_ and *U*

A suboptimal approach to estimating the value of the dynamic treatment decisions in the example two-stage scenario might be to specify an ‘all-at-once’ regression model for the outcome *Y* as a function of all the covariates, treatments, and various interactions among them, and to find the treatments *a*_1_ and *a*_2_ that optimise the expected value of *Y* (perhaps conditional on values of *o*_1_ and *o*_2_) [8]. As appealingly simple as the ‘all-at-once’ approach may seem, it may result in poor treatment decisions because the causal effects of treatment are improperly estimated for the following reasons:

- ▪ The effect of *A*_1_ on *Y* can be decomposed into direct and indirect effects. If *O*_2_ is a ‘child’ of *A*_1_ (i.e., the value of *O*_2_ is influenced by *A*_1_), including *O*_2_ (a treatment-outcome confounder) as a model covariate blocks the indirect effect of *A*_1_ on *Y*, as seen in Fig. 3, attenuating the estimated treatment effect of *A*_1_. In the language of causal inference, we say that *O*_2_ mediates the effect of *A*_1_ on *Y*.
- ▪ Even if *O*_2_ were not a mediator of *A*_1_, or treatment (*A*_2_)-outcome (*Y*) confounder, including *O*_2_ as a model covariate could induce collider stratification bias in the presence of unmeasured covariate (*O*_2_)-outcome confounders (*Y*) as seen in Fig. 4.

Because standard regression methods fail to account for the complexities inherent in DTRs, more sophisticated statistical methods are required. The exact methodology employed often depends on, and is tailored to, the clinical question of interest. The typical approach is to specify and estimate either a *dynamic conditional model* or a *dynamic marginal structural model* (MSM).

A dynamic conditional model defines the average effects of treatments conditional on patient history as target parameters for estimation. The estimated effects can therefore be considered to be personalised in that it is defined only for patients who have the same histories. To account for the effect mediation and biases depicted in Figs. 3 and 4, dynamic conditional models typically specify the treatment effects on a stage-by-stage basis. Estimating the treatment effects in dynamic conditional models often proceeds using *Q-learning* [19, 20], the *parametric G-formula* [1, 14, 21], or *G-estimation* [3, 22].

A dynamic MSM defines the average treatment effects of following different regimens as the target parameters for estimation. Key to this approach is identifying that many individuals will have histories that are, at least in part, compatible with several regimens. Approaches that use dynamic MSMs rely on creating, for each candidate regimen, replicates of the original data where individuals are artificially censored if they no longer follow the candidate regimen and aim to estimate the treatment effect of the candidate regimen while balancing prognostic factors among the treatment groups using inverse probability weighting (IPW) [1, 4, 14, 23, 24].

Although estimation methods such as Q-learning or IPW typically use relatively simple generalised linear models (for example linear and logistic regression), other estimation methods using the parametric G-formula or G-estimation may require complex estimating equations and/or large sets of models. In all cases, estimation performance can be sensitive to model misspecification, particularly when using the parametric G-formula which tends to use many interrelated models [1]. Although bias can be minimised through the use of ‘doubly-robust’ estimators—which produce unbiased estimates if at least one of the treatment or outcome submodels is correctly specified—there are efficiency gains to be made when both submodels are correctly specified [1]. Therefore, principled model selection, evaluation, and sensitivity analysis methods are highly recommended to mitigate the risk of model misspecification. Furthermore, as with most longitudinal data, missingness is often an additional source of bias, and principled approaches to handle missing data should also be used.

#### Causal assumptions

Several conditions must be met for the estimated DTR effects to have a causal interpretation [1, 14]. This is true whether using data from studies with a SMART design or observational data. Broadly, the key necessary conditions for causal inference can be summarised as *exchangeability*, *consistency*, and *positivity*. These conditions require that there are no unmeasured confounders (exchangeability), well-defined treatments (consistency), and that the probability of receiving each treatment regimen of interest is greater than zero for each patient included in the analysis (positivity). A complete and rigorous description of these assumptions is beyond the scope of this review, however Hernán and Robins [14] provide an accessible explanation of these conditions, and Chakraborty and Moodie [1] formalise each condition in the context of DTRs.

## Methods

### Protocol

The review protocol was developed by RKM and JAS in consultation with the co-authors. The original version of the protocol, along with the changes to the protocol, is available as an additional file (see Additional file 1).

### Eligibility criteria

To be included in the review, studies must have used statistical methods to estimate the value of DTR decision rules from observational data, either as a demonstration of the methodology or to provide real-world evidence to support specific treatment policies. *Statistical methods* were defined in this context as any method that fits a parametric, semi-parametric, or non-parametric statistical model to data using methods such as maximum likelihood estimation or estimating equations. This definition was broad enough to encompass most conceivable data analytical methods, including methods that are traditionally less aligned with biostatistical and epidemiological fields (for example, methods using artificial intelligence). *Observational data* were defined as any non-simulated data where the treatments of interest were not randomly allocated. No restriction was placed on study time period, publication type, statistical method, outcome types, sample size, country of origin, or participant characteristics.

Studies were excluded from this review if they met any of the following criteria:

- ▪ only analysed data from experimental studies where the treatment/s were randomised (including SMART designs and other randomised trials),
- ▪ analysed simulated data or provided theoretical discussion only,
- ▪ provided a commentary, review, opinion, protocol, or description only,
- ▪ either the abstract or full-text were not available,
- ▪ analysed data from non-human subjects only,
- ▪ studies were not available in the English language, or
- ▪ did not use statistical methods to evaluate a DTR using observational data, for example provided only a graphical or textual description of the data.

### Information sources

To identify potentially relevant studies the electronic bibliographic database PubMed was searched on 8 October 2020. The reference lists of the included articles identified from the PubMed database were manually screened to identify additional relevant studies. Grey literature, unindexed journals, and trial registries were not searched.

### Search strategy

The search strategy was developed by RM and JAS, with input from all co-authors, and in consultation with the University of Melbourne Library. The electronic PubMed search strategy is described in Table 1.

**Table 1:** PubMed search terms

Search Term Number	Search Term
1	dynamic treatment*[tiab]
2	adaptive treatment*[tiab]
3	dynamic intervention*[tiab]
4	adaptive intervention*[tiab]
5	treatment policy [tiab] OR treatment policies [tiab]
6	adapt*[tiab] OR dynamic*[tiab] OR regime*[tiab]
7	register [tiab] OR registry [tiab] OR registries [tiab] OR observational [tiab] OR cohort [tiab] OR non-experimental*[tiab] OR real-world [tiab] OR database [tiab] OR electronic health record*[tiab] OR electronic medical record*[tiab] OR non-randomised [tiab] OR panel [tiab] OR cross-sectional [tiab] OR longitudinal [tiab] OR case series [tiab])
8	(1 OR 2 OR 3 OR 4 OR (5 AND 6)) AND 7

Note: [tiab] indicates that the search was within article titles and abstracts only

### Selection of sources of evidence

RKM performed the search of the PubMed database, screened the titles and abstracts returned by the search, and reviewed the full text of all potentially eligible studies that satisfied the selection criteria for eligibility. Excluded studies were categorised by primary reason for exclusion. Titles and abstracts from each bibliography item of the included PubMed articles were also screened (not including books/book chapters, clinical guidelines, in proceedings, manuals/technical reports, software, posters, in press/submitted, theses, trial registries, or working papers), and all studies that satisfied the selection criteria for eligibility were included in the data synthesis.

### Data items

The data extracted from each article included reference details, study characteristics, data type, statistical methods, and whether the study was primarily intended to inform statistical or clinical practice (as defined below, see Table 2). The data extraction items were initially piloted by RKM and JAS for a subset of six articles and refined in consultation with the co-authors. Note that a methodological study typically aims to extend an existing method, present a novel method, or demonstrate the application of an existing method in a novel way. These studies typically involve a precise mathematical description of the method under investigation, demonstration of the statistical properties of the method either analytically or using computer simulation, and often include a highly stylised application of the method with real-world data. In contrast, a clinical study applying a statistical method to investigate a clinical research question typically involves collecting real-world data (either prospectively or retrospectivity), applying a validated statistical method to the data to address the clinical research question, and interpreting the results in a way that they might be used to inform either clinical practice or future clinical research. Although the boundary between clinical and methodological studies is at times unclear, in general, the category a study belongs to can be clearly identified by its aims, journal, mathematical density, and tone of the discussion.

**Table 2:** Data extraction items

Data	Definition^a^
Complete reference	Title, publication source, authorship, year published
Clinical area	Disease or medical condition studied, e.g., HIV/AIDS, cancer.
Outcome type	Type of primary outcome, e.g., binary, continuous, time-to-event.
Participants	Number of study participants included in the model (largest number if multiple analyses were performed).
Funding source/s	What direct funding sources were acknowledged? E.g., public, non-profit, industry-sponsored, not funded, not reported.
Statistical method/s	The statistical method/s used to estimate the value of the dynamic treatment regimen/s decision rules, e.g., inverse probability weighting, parametric G-formula, Q-learning.
Clinical focus	Was the main discussion and methodology of the study focused on directly informing clinical practice, or developing and evaluating a statistical method to answer a medical question?
Missing data	Were methods used to account for missing data included, e.g., multiple imputation, last observation carried forward, complete case analysis. Note: applies only to original data, not augmented data?
Model evaluation	Were methods used to evaluate the estimated model included, e.g., cross-validation, Bayesian information criterion, residual analysis?
Covariate selection	Was the approach for selecting the covariates stated, e.g., stepwise selection, convenience, subject matter expertise, causal directed acyclic graph, or analogous method?
Sensitivity analysis	Was model sensitivity assessed and how this was performed included, e.g., alternative model specification, truncated inverse probability weights?
Software included	If any analysis software code was included, what language was it written in, e.g., R, SAS, Python, Stata?

^a^If multiple models evaluated, definitions relate to dynamic treatment regimen models only

### Data extraction

Data on the fields listed in Table 1 were extracted using a standardised form (in Microsoft Excel) for each article by RKM and confirmed by a second reviewer (JAS or MM) for approximately 10% of the included articles. Any differences in extracted data fields were resolved by consensus between RKM and the second reviewer.

### Synthesis of results

The extracted data was explored using narrative synthesis and summarised using descriptive statistics. Studies were compared between subgroups defined by the primary focus of the study (clinical vs methodological). All data management and analysis was performed using the R programming language [25].

## Results

**Fig. 5:**
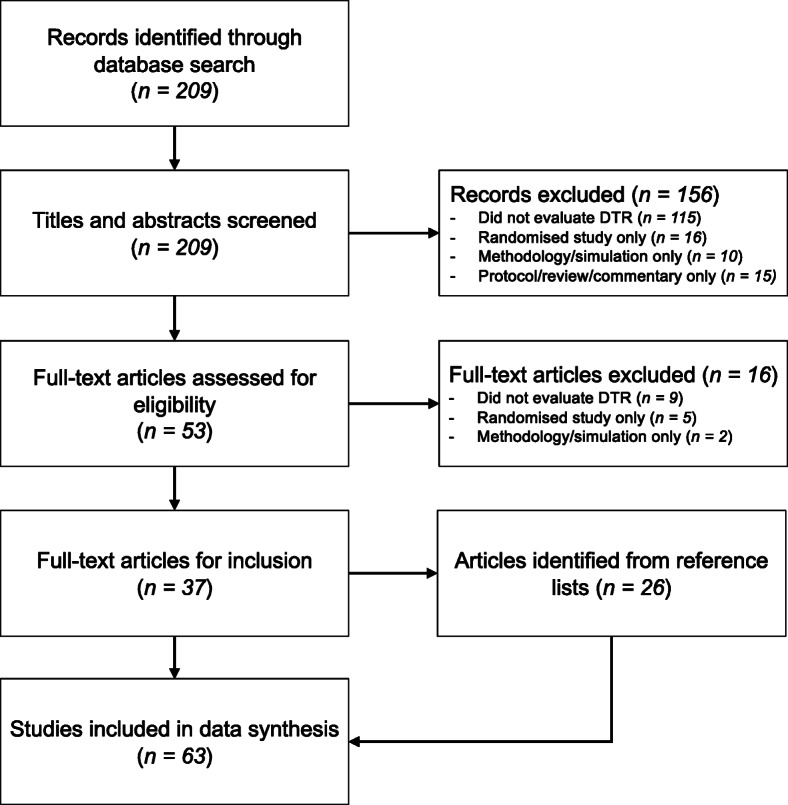
Search strategy flowchart. Note: *DTR* dynamic treatment regimen

The initial search returned 209 studies. Of these, 156 (75%) were excluded following screening of titles and abstracts. Upon reviewing the full-texts for eligibility, 37 studies were included from the PubMed database and a further 26 studies were identified from the PubMed article reference lists. In total, 63 studies were included in the data synthesis [3, 4, 6–9, 24, 26–81]. The flow chart of study selection is presented in Fig. 5. A summary of the data synthesis is provided in Table 3, and the extracted data for each study are provided in an additional file (see Additional file 2). Of the seven included studies which were reviewed by a second author, there was disagreement on a single data item that was resolved by consensus.

**Table 3:** Descriptive summary of the characteristics of the included studies

	Clinical focus		
Characteristic	No	Yes	Overall
Publications (n)	45	18	63
Year published (%)			
1995 to 1999	1 (2)	0 (0)	1 (2)
2000 to 2004	2 (4)	0 (0)	2 (3)
2005 to 2009	6 (13)	3 (17)	9 (14)
2010 to 2014	12 (27)	6 (33)	18 (29)
2015 to 2019	18 (40)	7 (39)	25 (40)
2020	6 (13)	2 (11)	8 (13)
Clinical area (%)			
Cancer	7 (16)	1 (6)	8 (13)
Diabetes	6 (13)	0 (0)	6 (10)
HIV/AIDS	15 (33)	12 (67)	27 (43)
Other	14 (31)	5 (28)	19 (30)
Thrombosis	3 (7)	0 (0)	3 (5)
Outcome type (%)			
Binary	4 (9)	3 (17)	7 (11)
Categorical	1 (2)	0 (0)	1 (2)
Continuous	17 (38)	2 (11)	19 (30)
Time-to-event	23 (51)	13 (72)	36 (57)
Participants (median [IQR])	2604 [710, 13,039]	9793 [3084, 39,887]	3882 [1420, 23,602]
Funding source/s (%)			
Non-profit	4 (9)	2 (11)	6 (10)
Not reported	9 (20)	1 (6)	10 (16)
Public	35 (78)	16 (89)	51 (81)
Statistical method/s (%)			
G-estimation	4 (9)	0 (0)	4 (6)
Inverse probability weighting	17 (38)	9 (50)	26 (41)
Other	9 (20)	1 (6)	10 (16)
Parametric G-formula	8 (18)	8 (44)	16 (25)
Q-learning	9 (20)	1 (6)	10 (16)
Regret regression	3 (7)	0 (0)	3 (5)
TMLE	4 (9)	0 (0)	4 (6)
Missing data addressed (%)	24 (53)	8 (44)	32 (51)
Model evaluation included (%)	22 (49)	8 (44)	30 (48)
Model selection included (%)	19 (42)	15 (83)	34 (54)
Sensitivity analysis included (%)	23 (51)	15 (83)	38 (60)
Software included (%)			
No	25 (56)	17 (94)	42 (67)
R	18 (40)	0 (0)	18 (29)
SAS	2 (4)	1 (6)	3 (5)

Abbreviations: *AIDS* Acquired immune deficiency syndrome, *HIV* Human immunodeficiency virus, *IQR* Inter-quartile range, *TMLE* Targeted maximum likelihood (minimum loss-based) estimation. Note that each study could have multiple funding sources and statistical methods. Percentages are rounded and taken with respect to number of publications in each column

The estimation of optimal DTRs using observational data is a recent development and has been most concentrated in in the area of HIV/AIDS (27, 43%), followed by cancer (8, 13%), and diabetes (6, 10%). All but three of the included studies were published after 2005, with all but nine in the last decade and almost half (25, 45%) in the last 5 years.

Outcome types, participant numbers, and funding sources varied considerably between the included studies. Time-to-event outcomes were most commonly investigated (36, 57%). The median number of participants was 3882 with an interquartile range (IQR) between 1420 and 23,602, and the total range between 133 and 218,217. Studies were funded mostly through public sources (51, 81%), with some studies acknowledging non-profit sources (6, 10%). Ten (16%) studies did not report on funding sources.

All of the common statistical approaches that we have described were implemented, yet there was a lack of transparency regarding some of the specific methodological approaches used across many studies. IPW-related methods were the most commonly used (26, 41%), followed by parametric G-formula related methods (16, 25%), Q-learning related methods (10, 16%), G-estimation (4, 6%), targeted maximum likelihood/minimum loss-based estimation (4, 6%), regret regression (3, 5%), and other less common approaches (10, 16%). Many studies did not clearly and explicitly describe the methods that they employed for either missing data (32, 51%), model evaluation (30, 48%), model selection (34, 54%), or model sensitivity (38, 60%), and only eight studies described all four methodological approaches. The studies that published statistical software code relevant to their analyses (21, 33%) provided it for either R (18, 29%) or SAS (3, 5%) only.

Eighteen (29%) studies had a clear primary focus of informing clinical practice. The remaining 45 (71%) of studies used observational data only to illustrate the application of statistical methodology [40, 79]. The median sample size of clinical studies was 9793 participants (IQR: 3084, 39,887), considerably higher than that of methodological studies (median: 2604, IQR: 710, 13,039). Compared to methodological studies, clinical studies were likely to focus on HIV/AIDS (12, 67% vs 15, 33%), time-to-event outcomes (13, 72% vs 23, 51%), and statistical models that used IPW (9, 50% vs 17, 38%) or the parametric G-formula (8, 44% vs 8, 18%). Although methodological and clinical studies described missing data and model evaluation methods in approximately equal proportions, a much greater proportion of clinical studies described their methods for model selection (15, 83% vs 19, 42%) and sensitivity analysis (15, 83% vs 23, 51%). Only one clinical study included statistical computing code used for analysis.

## Discussion

This review provided a summary of how DTRs can be modelled and an overview of how observational data have been used to estimate optimal DTRs. There was substantial variation in the scope, intent, complexity, quality, and statistical methodology between the 63 included studies.

DTR models are often necessary when formalising decisions about how best to treat chronic or progressive conditions to properly account for time-varying treatment confounding and mediation. A number of different statistical approaches can be used—including IPW, Q-learning, the parametric G-formula, G-estimation, or targeted maximum likelihood/minimum-loss based estimation—depending on the DTR model used and the nature of the research question. Almost all clinical studies used either IPW or the parametric G-formula methods, possibly because these methods are relatively well-established, less complex, and suited to simpler decision problems such as those encountered in HIV/AIDS treatment. Unsurprisingly, the included methodological studies were more diverse in the methods that they used and tended to detail model selection and sensitivity analyses less often. Encouragingly, this review found that many included studies often dealt with clinically relevant but complicated time-to-event outcomes.

Evaluation of dynamic treatment regimens was first described in 1987 by Robins [21], but most of the studies included in this review were published in the last 10 to 15 years, perhaps because both the methodology has matured and formal causal inference methods in epidemiology have become more established. Two-thirds of the clinical studies and one-third of methodological studies focused on HIV/AIDS, most likely because of the chronic and progressive nature of HIV infection and AIDS for which treatments are often dynamically, if informally, adapted to patient history.

Compared to randomised controlled trials, using observational data to estimate DTRs may allow researchers to both take advantage of the economics of using existing data and also evaluate a wider range of treatments. Despite this, the majority of included studies did not have a clinical focus. Of the clinical studies, most focused on HIV/AIDS, and analysed large datasets using either IPW or parametric G-formula methods to answer relatively simple questions. This result provides insight into the type and scale of resources, and research questions, that may give rise to feasible observational DTR studies.

The majority of studies were methodological investigations and typically included a simplified real-world application only. Many of the included methodological articles involved methods and results that were based on complex estimating equations and/or Monte Carlo simulations which, although no doubt critical for the advancement of the DTR methodology, may be difficult for clinical readers to interpret. It is likely that user-friendly software would make implementing the complex methods easier for clinicians and methodologists alike. Although almost half of the methodological studies included some form of statistical software code related to their methods, which may encourage the application of complex DTR methods, in general this software is not readily usable by non-experts. Furthermore, many studies did not describe the real-world applications or include details of the statistical methods and corresponding assumptions in detail, which may limit how the DTR methods and results are translated in practice.

We posit that the limited number of clinically relevant examples of optimised DTRs using observational data is because of the need to satisfy three conditions necessary for estimating causal treatment effects: exchangeability, positivity, and consistency. These conditions, required for valid causal inference, cannot be verified from the data alone and require judgement on biological plausibility.

To meet the exchangeability condition, explicit causal relationships must be considered by content experts to identify confounders and the confounder data must be available. Developing a causal model requires both clinical expertise and statistical knowledge to codify such expertise using the causal inference framework. Although the use of DAGs can streamline this process, it still requires substantial investment in learning and collaboration by both content experts and data analysts, particularly if multiple plausible causal models are developed to assess sensitivity of conclusions. Even when it is feasible to fully develop a plausible set of causal models it is not guaranteed that confounder information will be available, particularly when working with retrospectively collected data or electronic health records, which are often designed around clinical practice rather than for research purposes. It is worth noting that fewer than 50% of studies did not describe the model selection process in any way.

To ensure causal effects can be estimated, the positivity condition must be met. This requires that all regimens of interest are followed by at least some (and, in practical terms, many) patients for each potential combination of predictors and outcomes. Large clinical databases, and questions about non-rare medical conditions, are likely to be required for there to be sufficient numbers such that the positivity condition holds. We note that many of the clinical studies that we identified in this review used either very large EHR databases or data from large multinational collaborations, and focused on a relatively prevalent medical condition. Even with large clinical databases, structural factors such as clinical, regulatory, or reimbursement guidelines may completely prevent treatment sequences of interest (not to mention relevant patient histories) from being observed.

The consistency condition requires that treatments, and therefore potential outcomes under treatments, are sufficiently well-defined, which may be a difficult condition to meet for conditions where there are many different treatment modalities. A related point is that in clinical areas with rapid and continual treatment innovation the clinical paradigm may change so rapidly that DTRs modelled using data from observational cohort studies or EHRs, with patient treatment histories over a long time period, are less relevant to informing clinical practice. For example, management of many cancers often involves several consecutive lines of treatment following disease progression and determining the optimal sequence of treatments is an open area of research in modern oncology. But new cancer treatments and changing clinical paradigms often dramatically change the treatment landscape, which results in substantial variation in clinical practice. Over time, treatments become less well-defined, and it becomes difficult to satisfy the consistency condition.

Although we are satisfied that our scoping review provides a representative sample of the literature there are some limitations worth noting. Our exclusive focus on the PubMed database excludes any studies not indexed therein. We made this choice early on in the design process on the basis of our broad aims, the ‘scoping’ nature of our review, and also to simplify the review and make it as reproducible and transparent as possible. We note that searching the reference lists of the included PubMed articles served as a practical workaround of the limitation arising from using a single database. Further, the search strategy included only common phrases, and their variants, to capture both DTRs and observational data. There may be variants that we have missed, or there may be ad hoc implementations that use entirely different naming conventions or combinations thereof, although we note that the nomenclature concerning dynamic treatment regimens is relatively well-established in the literature.

## Conclusions

Using observational data to model DTRs is a modern and methodologically principled approach to evaluating dynamic treatment decisions. There is great potential in using DTR models with existing observational data to support dynamic treatment decisions that improve patient outcomes, particularly where the relevant clinical trial is not feasible. Yet the use of observational DTR studies to inform clinical practice has been relatively limited, primarily because the underlying conditions that are necessary for causal inference are difficult to satisfy. Developing new methods that enable these conditions to be satisfied may more broadly enable additional and more diverse observational DTR studies. Our review suggests that the currently available methods are most likely to find feasible applications for relatively simple dynamic clinical decisions, either for simple treatment sequences or ‘when to treat’ type questions, where there are numerous and rich clinical data, where treatments can be well-defined, in clinical areas with slowly evolving treatment paradigms, and where content experts and data analysts work in tight partnership.

## Supplementary Information

**Additional file 1.** Original scoping review protocol.

**Additional file 2.** Extracted data for individual studies.

## Data Availability

The datasets used and analysed during the current study are available from the corresponding author on reasonable request.

## Abbreviations

AIDS: Acquired immune deficiency syndrome
DAG: Directed acyclic graph
DTR: Dynamic treatment regimen
HIV: Human immunodeficiency virus
IPW: Inverse probability weighted
IQR: Interquartile range
MSM: Marginal structural model
SMART: Sequential multiple assignment randomised trial
TMLE: Targeted maximum-likelihood/minimum-loss based estimation
