# Supplementary material for: A scoping review of studies using observational data to optimise dynamic treatment regimens
Source: BMC Med Res Methodol. 2021 Feb 22;21:39. doi: 10.1186/s12874-021-01211-2 (PMC7898728; doi:10.1186/s12874-021-01211-2)
Supplement: Supplementary file 1 — Additional file 1. Original scoping review protocol. [file 12874_2021_1211_MOESM1_ESM.pdf]

Optimising dynamic treatment regimens using observational data: a scoping review.

Review protocol

Version 0.1

Contents

Version history **3**

Preface **4**

1 Review question **5**

2 Search strategy **5**

2.1 Types of study to be included 5

2.1.1 Inclusion criteria 5

2.1.2 Exclusion criteria 5

2.2 Condition or domain 6

2.3 Participants 6

2.4 Interventions/exposures 6

3 Data extraction **6**

4 Strategy for data synthesis **7**

5 Review member and affiliations **7**

6 Anticipated or actual start date **8**

7 Anticipated completion date **8**

8 Funding sources/sponsors **8**

9 Conflicts of interest **8**

10 Country **8**

11 Tables **9**

Version history

Version	Date	Author	Description
0.1	21 Nov 2019	Robert Mahar	Initial draft

Preface

This scoping review protocol was initially drafted on 21 Nov 2019, after piloting a number of approaches among the authorship group. Since then the scoping review has undergone several revisions at the request of reviewers. The present protocol was not published and therefore the details of the revised review were simply included in an updated main manuscript rather than an updated protocol, however we have included the original protocol for completeness. Note the following key changes between this protocol and the updated review as it appears in the main manuscript:

- We expanded the search strategy to include screening the titles and abstracts of journal articles that were cited in the reference lists of the PubMed articles that were included for data synthesis.
- We no longer included any data that originated from randomised trials, even if the data were analysed in an 'observational' way, for example an 'as treated' analysis. We felt that focusing on non-trial data only provided more meaningful results that were in line with the aims of the review i.e. how dynamic treatment regimens are optimised using observational data, thus there were no exceptions to the 'experimental' exclusion criteria (see Section 2).
- We opted not to categorise the source of 'data type' (see Table 2) used by each study for two reasons: (1) excluding trials made the categorisation less important, and (2) upon updating the review the source of the observational data for many papers was not clearly defined. Instead, we have chosen to focus on the participant numbers available for analysis along with a more narrative synthesis.
- The updated review was considerably larger, and therefore the proportion of articles included in the subset that was validated by a second reviewer is now 1/10, rather than 1/3 as stated in Section 3.
- Other changes, not specified, such as minor changes to wording and structure, and inclusion of additional detail, to improve interpretation of the methods and results.

For further detail on how the updated review was conducted, please refer to the main manuscript.

1 Review question

What types of data and statistical methods are used by studies that estimate optimal dynamic treatment regimens from observational data? Do these studies adequately describe key methodological aspects of their analysis? Did the study appear to be designed more to inform statistical or clinical practice?

2 Search strategy

The electronic bibliometric database PubMed will be searched using a search strategy based on whether the title or abstract included the terms synonymous with dynamic treatment regimens, for example 'adaptive treatment strategies'.. Primary search terms constructed as an ordered combination of 'dynamic', 'adaptive', and 'treatment', 'intervention' in the title or abstract. The full search strategy is outlined in Table 1.

2.1 Types of study to be included

2.1.1 Inclusion criteria

Studies will be included in the review if they used statistical methods to model dynamic treatment decisions from observational data, either as a demonstration of the methodology or to provide real-world evidence to support specific treatment decisions. Statistical methods are defined in this context as any method that estimates a parametric, semi-parametric, or non-parametric statistical model using data. Observational data is defined as any data where the treatment/s of interest were not randomly allocated. No restriction is placed on time period, publication type, participant characteristics,

2.1.2 Exclusion criteria

Studies were excluded from the review if they met the any of following criteria:

- Experimental: analysed data from experimental studies only (including SMART designs and other randomised trials) with the exception of trial data analysed according to treatment received (i.e.

treatment assignment subject to confounding).

- Methodological: analysed simulated data or provided theoretical discussion only (whether experimental or non-experimental data were used).
- Qualitative: provided a commentary, review, opinion, or description only (whether experimental or non-experimental data were used).
- No text or abstract: did not provide either an abstract or full-text.
- Non-human subjects: analysed data from non-human subjects only.
- Language other than English: studies were not available in the English language.
- Not relevant: were otherwise not relevant to the statistical modelling and estimation of real-world dynamic treatment regimens.

2.2 Condition or domain

All disease areas/medical conditions will be considered.

2.3 Participants

The review is restricted to studies of human populations. No restrictions as to age or ethnicity were considered.

2.4 Interventions/exposures

Dynamic treatment regimens.

3 Data extraction

The search of the PubMed database will be performed by a single researcher using PubMed's application programming interface implemented from an R programming environment. The titles and abstracts of

publications returned by the database search will automatically be exported to Excel and screened by a single researcher for eligibility. All potentially eligible studies will be reviewed, and main outcomes extracted, by a single researcher. Another researcher will confirm the accuracy of the extracted outcomes for a random subset of 1/3 of the eligible studies. Disagreements will be resolved by consensus between the two disagreeing researchers. The main outcome data to be extracted is described in Table 2.

4 Strategy for data synthesis

The extracted data will be imported from Excel into an R programming environment. Qualitative data will be synthesised in a narrative format. No minimum number of studies is required for any of the outcome measures.

5 Review member and affiliations

Dr Robert K Mahar[1,2,3], Dr Myra B MacGuiness[1,4], A/Prof Bibhas Chakraborty[5,6,7], Prof John B Carlin[1,8], Prof Maarten J Ijzerman [2,3,9], Prof Julie A Simpson[1]

1. Biostatistics Unit, Centre for Epidemiology and Biostatistics, Melbourne School of Population and Global Health, University of Melbourne, Parkville, Victoria, Australia
2. Cancer Health Services Research Unit, University of Melbourne Centre for Cancer Research and Centre for Health Policy, Melbourne School of Population and Global Health, University of Melbourne, Parkville, Victoria, Australia
3. Victorian Comprehensive Cancer Centre, Parkville, Victoria, Australia
4. Centre for Eye Research Australia, Royal Victorian Eye and Ear Hospital, Melbourne, Victoria, Australia
5. Centre for Quantitative Medicine, Duke-NUS Medical School, Singapore
6. Department of Statistics and Applied Probability, Faculty of Science, National University of Singapore, Singapore

7. Department of Biostatistics and Bioinformatics, Duke University, Durham, North Carolina, United States of America

8. Clinical Epidemiology and Biostatistics Unit, Murdoch Children's Research Institute, Parkville, Victoria, Australia

9. Peter MacCallum Cancer Centre, Parkville, Victoria, Australia

Corresponding author: Robert Mahar, Melbourne School of Population and Global Health, 207 Bouverie Street, University of Melbourne, Carlton, Victoria, 3053, Australia. robert.mahar@unimelb.edu.au

6 Anticipated or actual start date

1 April 2019

7 Anticipated completion date

1 Feb 2020

8 Funding sources/sponsors

Not applicable.

9 Conflicts of interest

None known.

10 Country

Australia, Singapore.

11 Tables

Table 1: PubMed search terms

**Search term number**	**Search term**
1	dynamic treatment*[tiab]
2	adaptive treatment*[tiab]
3	dynamic intervention*[tiab]
4	adaptive intervention*[tiab]
5	treatment policy[tiab] OR treatment policies[tiab]
6	adapt*[tiab] OR dynamic*[tiab] OR regime*[tiab]
7	register[tiab] OR registry[tiab] OR registries[tiab] OR observational[tiab] OR cohort[tiab] OR non-experimental*[tiab] OR real-world[tiab] OR database[tiab] OR electronic health record*[tiab] OR electronic medical record*[tiab] OR non-randomised[tiab] OR panel[tiab] OR cross-sectional[tiab] OR longitudinal[tiab] OR case series[tiab])
8	(1 OR 2 OR 3 OR 4 OR (5 AND 6)) AND 7

Note: '[tiab]' indicates that the search is conducted on article titles and abstracts only.

Table 2: Data extraction details

**Search term number**	**Search term**
Complete reference	Title, publication source, authorship, year published.
Clinical area	Disease or medical condition study focused on: e.g., HIV/AIDS, cancer.
Data type	Type of data used: e.g., cohort study, trial, electronic health record (including administrative, hospital, and governmental data).
Outcome type	Type of primary outcome: e.g., binary, continuous, time-to-event.
Participants	Number of study participants included in the model (largest if multiple analyses).
Model	The statistical model/s that used to model the dynamic treatment regimen: e.g., marginal structural model, parametric formula.
Missing data	The method/s that were used to account for missing data: e.g, multiple imputation, last observation carried forward, complete case analysis.
Model evaluation	The method/s that were used to evaluate the estimated model: e.g.,
Covariate selection	The method/s that were used to select the covariates: e.g., cross-validation, Bayesian information criterion, stepwise selection.
Sensitivity analysis	The method/s that were used to assess model sensitivity: e.g., changing model specification.
Clinical design	Was the study was designed specifically to inform clinical practice.
Software included	If any source code was included, what language was it written in: e.g., R, SAS, Python, Stata.

HIV/AIDS: human immunodeficiency virus/acquired immunodeficiency syndrome
